# Changing trajectory of daily physical activity levels among at-risk adolescents: influences of motivational mechanisms

**DOI:** 10.1186/s12889-023-16949-1

**Published:** 2023-10-25

**Authors:** Anqi Deng, Nicole Zarrett, Jongho Moon, Allison M. Sweeney

**Affiliations:** 1 Department of Psychology, University of South Carolina, Columbia, USA; 2 Behavioral Medicine Group, Department of Psychology, College of Arts and Sciences, University of South Carolina, 1330 Lady Street, Suite 400, Columbia, SC 29201 USA; 3 Department of Biobehavioral and Nursing Science, College of Nursing, University of South Carolina, Columbia, USA

**Keywords:** Moderate to vigorous physical activity, Community based intervention, Health disparities, Minorities, Intrinsic motivation

## Abstract

**Background:**

Guided by Self-Determination Theory (SDT), the purpose of this study was to determine changes in the 16-week moderate-to-vigorous physical activity (MVPA) trajectory of underserved adolescents who participated in the Connect through PLAY afterschool program intervention and the effects of changes in participating adolescents’ intrinsic and autonomous extrinsic motivations on their MVPA trajectory over the 16-week intervention.

**Methods:**

A subsample of 113 adolescents (56.64% female; 61.06% African American; average age = 11.29) provided complete data throughout the 16-week intervention were examined. Adolescents’ objective daily MVPA was measured using 7- day accelerometer data. Changes in adolescents’ intrinsic motivation and autonomous extrinsic motivation were assessed using subscales from the Intrinsic Motivation Inventory [1] and the Treatment Self-Regulation Questionnaire [2] respectively. A hierarchical linear model was built and tested to address the research aims.

**Results:**

The results of hierarchical linear models showed that, on average, youth daily MVPA increased 6.36 minutes in each 8-week period. Intrinsic motivation change, but not autonomous extrinsic motivation, was a positive and significant level-2 predictor of daily MVPA changes.

**Conclusion:**

The findings provide significant evidence suggesting a benefit of integrating SDT-based approaches and further suggest that nurturing intrinsic motivation can be an effective approach to supporting youth daily MVPA in under-resourced afterschool programs.

**Trial registration:**

Connect Through PLAY: A Staff-based Physical Activity Intervention for Middle School Youth (Connect). https://clinicaltrials.gov/ct2/show/NCT03732144. Registered November 6^th^, 2018.

## Introduction

Obesity and related chronic health issues affect the lives of many adolescents in the United States, partially due to chronically low rates of daily physical activity [1]. Daily physical activity (PA) is a key factor of sustaining good health and can improve physical, psychological, and social well-being [2]. Early-to-middle adolescence (ages between 9 to 15 years old) has been identified as a particularly important developmental period to promote motivation and engagement in PA as it is during this time that individuals establish long-term patterns of healthy behaviors and lifestyle choices that promote sustained physical activity, health, and wellbeing through adulthood [3, 4]. However, recent national studies indicate that only approximately 24% of children aged 6-17 years participate in 60 minutes of moderate-to-vigorous physical activity (MVPA) each day and significantly fewer adolescents (i.e., 7.5% of 12-15-year-olds, and 5.1% of 16-19-year-olds) meet MVPA recommendations compared to that of younger children (42.5% of 6-11-year-olds) [5]. The situation is particularly dire for adolescents from underserved, minority communities, as they face additional barriers, including lack of access to PA supportive practices, expensive “pay-to-play” recreational activity models, facility and equipment shortages, and high teacher or staff burn out rate [6]. Consequently, underserved minority youth have been consistently identified as having the lowest rates of PA [6] and the greatest need for PA intervention.

Afterschool programs (ASPs) are considered highly feasible sites for PA promotion and commonly offer various PA programs and opportunities that can be used to supplement PA time for adolescents [7]. Recent data indicates that over 7.8 million children and adolescents in the United States participate in ASPs that support healthy eating and PA opportunities [5] and have notable reach to minority youth within underserved communities [8]. ASPs can promote levels of daily PA time among youth and can also benefit them both socially and mentally [9]. Drawing from research on high quality physical education (PE) classroom instruction [10], researchers have begun to emphasize the need to foster a positive motivational climate as central to their programming to enhance youth’s meaningful and physically active learning experiences within ASPs [2, 8]. Several studies support that addressing social-motivational climate factors within ASPs could directly and/or indirectly, through intrapersonal motivational factors, influence adolescents’ PA participation [7, 9, 11, 12]. However, few studies have examined the key mechanisms through which a positive motivational climate intervention can support increases in adolescent MVPA. Informed by the Basic Needs Theory of the Self-Determination Framework (BNT) [13], the Connect through Positive Leisure Activities for Youth (PLAY) intervention is designed to establish an ASP climate that meets youth needs for autonomy, competence, and relatedness. Developmental and neurobiological research has indicated that biological and social-contextual developmental changes during early-to-middle adolescence (between the ages of 9 to 15 years old), also heighten motivational needs for social connections, meaningful interpersonal relationships, and the need for self-preservation within social contexts [14, 15]. Therefore, the Connect through PLAY intervention aims to meet youth basic needs for PA through fostering strong connections with peers and program staff, defining achievement (competence) through meeting social affiliation goals for PA [16, 17], and providing daily activity opportunities where all individuals have input/ownership over program components (autonomy) and feel they are valuable contributors to the group (relatedness) [8, 11]. In turn, settings that meet youth basic needs are expected to improve youth motivation and engagement in a program’s health initiatives/goals [18, 19]. However, youth motivation orientations are complex, with expected variations in the degree to which they are self-determined, and the nature in which they may change from a social-motivational climate intervention. As a key proposed mechanism of social-motivational climate interventions, it is critical to identify key motivational mechanisms, and their changes, that effectively promote PA in order to develop effective interventions among youth within underserved communities.

### Self-determination theory: key motivational mechanisms

According to Deci and Ryan [20], the need for autonomy, competence, and relatedness are three basic sources for human motivation, while satisfaction determines individuals’ degree of self-determined motivation. The need for autonomy refers to people’s desire to be the cause agent in their world [21]. If a behavior satisfies the need for autonomy, he or she will feel self-determined in his/her action rather than feeling controlled or obliged to act. The need for competence is satisfied through the pursuit of autonomously motivated behaviors that lead to perceptions of success and control of outcomes [21]. The relatedness need refers to the innate desire to be supported by or supportive to others when engaging in the activity [21]. Deci and Ryan [20] propose that the extent to which the three basic needs are satisfied determines the degree of autonomous extrinsic motivation people will experience when doing an activity. A key assumption of SDT is the notion that SDT-based climate supports are among the most significant elements influencing motivational processes, related behavioral outcomes, and the three basic psychological needs [9]. This includes the degree to which an individual is inherently or “intrinsically” motivated to engage in an activity or whether they are more extrinsically driven or completely amotivated to engage in the activity [21]. The motivational climate is substantially shaped by the nature of the activities/tasks offered (e.g., appropriate challenge, inclusive, providing choices) and the influence of other actors’ interpersonal activities within the setting (e.g., staff, teachers, peers, and parents) [9]. Integrating SDT-based climate supports through tailoring program activities to meet youth psychological needs and nurture positive interpersonal interactions, has been proposed to effectively promote motivation, efficacy, and engagement in PA [11, 21].

Motivation in SDT is conceptualized as different reasons that people engage in activities and the degrees to which an individual is inherently or “intrinsically” motivated to engage in an activity or whether they are more extrinsically driven or completely amotivated to engage in the activity [21]. Deci and Ryan [20] propose that amotivation, extrinsic motivation and intrinsic motivation form a continuum ranging from the lowest to the highest levels. Intrinsic motivation refers to a psychological state in which a person’s engagement in behaviors or activities is intrinsically regulated [20]. People who are intrinsically motivated toward an activity tend to engage in the activity for the sake of the activity itself and not as a means to achieve other internal or external purposes [20]. Extrinsic motivation, in contrast to intrinsic motivation, is a perception of a situation in which individuals engage in an activity to obtain certain desirable and separate outcomes. Based on the degree of self-determination, extrinsic motivation can be identified as controlled motivation (external and introjected regulations) or autonomous extrinsic motivation (integrated and identified regulations) [22]. Controlled motivation refers to behaviors that are not performed out of choice but are regulated by internal and/or external pressure [23] and research findings have consistently shown either negative or no significant effects on adolescents’ MVPA [24].

Autonomous extrinsic motivation, which is made up of integrated and identified regulations, is based on a sense of value – people view the activities as worthwhile, even if it’s not enjoyable [22, 25, 26]. It has been shown that the distinction between autonomous extrinsic motivation from that of controlled motivation and intrinsic motivation is conceptually and empirically important for PA behavior change [21, 22]. The two types of regulatory processes (i.e., identified and integrated) that define autonomous extrinsic motivation, are considered more internalized forms of extrinsic motivation. Identified regulation refers to individuals who perceive the activity as relevant to their personal goals or their self-concept and believe that the task is valuable [27]. For example, youth exercise because they appreciate the value that exercise can bring to their health. Integrated regulation is the highest or the most internalized form of extrinsic motivation [27]. At this level, the behavior is integrated into self, and becomes a part of the individual’s identity. The individual totally embraces the value of the task even though he or she may not enjoy the task. For example, youth exercise because exercise is important to them or supports their identity as an athlete [25].

### Physical activity motivation as a mechanism of behavior change

Research findings have identified intrinsic motivation as an important positive indicator of youth’s participation in PA across age groups [28, 29]. In cross-sectional studies, Kalajas-Tilga et al. [28] found that intrinsic motivation positively predicted MVPA among 297 adolescents (aged 11-15 years) from 17 schools. Sebire et al. [29] found that intrinsic motivation was positively associated with children’s MVPA (aged 7-11 years) from 20 elementary schools. However, studies examining the relationship between changes in intrinsic motivation and its longitudinal impact on changes in PA participation are limited. Only a single study has been centered on addressing motivational climates changes on adolescents’ PA behavior change. Lonsdale et al. [30] tested the effects of three motivational strategies (explaining the relevance of activities; providing choice from PA options selected by the teacher; providing equipment and free choice of activities) based on SDT in a cluster-randomized controlled trial project (Motivating Active Learning in PE) on 8^th^ graders’ PA levels in PE class. The results showed that although the intervention did not influence students’ SDT motivations, students’ PA and autonomy increased during the choice-based teaching strategies in the intervention group. However, few studies have considered these relations among youth from underserved minoritized communities.

Given the internalized nature of autonomous extrinsic motivation (i.e., integrated within one’s value system or self-system), it is expected that this form of motivation would support continued youth engagement in MVPA. Although studies examining intrinsic motivation have consistently shown positive relations with PA accrual [28–30], previous findings concerning the association between autonomous extrinsic motivation and PA are mixed. Fenton et al. [9] developed and tested a six-week ASP sports-based intervention and, using a path model, found an empowering motivational climate that initial autonomous extrinsic motivation positively predicted PA-related enjoyment, which was positively associated with daily MVPA among adolescents’ (aged 9-16 years). In contrast, Timo et al. [31], using a longitudinal research design, found that initial autonomous extrinsic motivation did not have a statistically significant role in predicting 6 year later adolescents’ MVPA in PE class. Although the discrepancies between studies may be explained by variations in research design (short-term vs longitudinal) as well as variations in PA contexts (ASP vs PE class), the current findings suggest that more research is needed to examine autonomous extrinsic motivation as another potentially effective motivational orientation that can be nurtured in youth to promote long-term increases in PA engagement.

### Gender and race as predictors

Physical activity participation varies significantly by youth gender and race/ethnicity due to differences in socialization experiences and access to contextual affordances and tangible and emotional supports (and barriers) in which youth have access depending on these ascribed characteristics [18, 32, 33]. These differences highlight the significant disparities in PA among girls and Black and Hispanic adolescents who are less likely to meet 60 minutes daily MVPA recommendations compared to boys and White youth [18, 34, 35]. In particular, girls worldwide consistently report significantly lower levels of PA than do boys and show steeper declines in PA from childhood through adolescence [36, 37]. In addition to the relationship between gender and physical activity behavior, Black and Hispanic adolescents report less physical activity than white adolescents [32]. Miller et al. [38] found that white adolescents reported 1.1 to 2.1 more weekly hours of MVPA than non-white adolescents by using self-reported MVPA measurement. Given gender and race have been reported as two important factors influencing SDT motivations and physical activity participation [32, 33], understanding how a PA social-motivational context intervention could impact participating youth differently by gender and race could guide future development of effective PA intervention programs.

In summary, two theoretical and empirical gaps in the SDT-related literature warrant the current study. First, although the correlational evidence on intrinsic motivation, autonomous extrinsic motivation, and adolescents’ MVPA have been established, there are few studies that examine changes in intrinsic motivation and autonomous extrinsic motivation resulting from participation in a social-motivational context intervention that supports adolescents’ need for autonomy, competence, and relatedness, and its impact on underserved adolescents’ daily MVPA change trajectory. Secondly, the effect of gender and race on at-risk adolescents’ changing daily MVPA trajectory are tested in this study given the significant disparities in PA observed among girls and Black and Hispanic youth. In summary, more studies are needed to further understand the role of self-determination motivations for increasing youth daily PA engagement, with particular attention to potential variations in these relations for youth who have been identified at greatest risk for inactivity, to inform the development of high-quality interventions within youth settings serving at-risk youth.

Guided by SDT, the purpose of this study was to determine the 16-week MVPA trajectory of underserved adolescents who participated in the Connect through PLAY intervention, whether there are variations in these trajectories by gender and race, and the effects of changes in adolescent intrinsic and autonomous extrinsic motivations over the 16-week intervention on the trajectory. Specifically, the following research questions were addressed in the current study: (a) to what extent does adolescents’ daily MVPA change from baseline to endpoint during a 16-week SDT-based intervention within ASPs, and does this change vary by gender and race? (b) to what extent does change in adolescent’s self-determined motivation (intrinsic and autonomous extrinsic motivation) across the 16-week intervention influence the 16-week trajectory of their daily MVPA time?

## Methods

### Participants

A total of 193 adolescents in the PA intervention arm (from five ASPs) participated in baseline data collection between 2018 to 2020 during Y1 and Y2 of the 5-year trial. The global coronavirus disease outbreak of 2019 (COVID-19) forced school/ASP closures during March of 2020 resulting in incomplete endpoint data collection in Y2 of the study. Therefore, for the current study, only the subsample of participants with complete data at baseline, midpoint, and endpoint were included in the analyses (*n* = 113). Chi-Square tests indicated that there were no significant differences for gender [*χ*^2^ (2, 193) = 5.52, *p* = .063], race [*χ*^*2*^ (3, 193) = 5.96, *p* = .11], and age [*χ*^*2*^ (6, 193) = 9.87, *p* = .13] between youth who were included in the data analysis and those were not included. In addition, results showed that there were no significant differences for intrinsic motivation [*χ*^*2*^ (14, 193) = 25.14, *p* = .33] and autonomous extrinsic motivation [*χ*^*2*^ (88, 193) = 89.84, *p* = .42] between adolescents who were included in the data analysis and those were not included. A total sample of 113 adolescents (56.64% female; average age = 11.29, Standard Deviation = 1.06) from five ASPs provided complete data throughout the 16-week intervention. Among these adolescents, the majority identified as African American (61.06%), 25.66% identified as European American, and 13.28% identified as biracial, Asian, Pacific Islander or other.

### Research design and data sources

The study was a part of a 5-year prospective randomized controlled trial intervention study implemented within 30 pre-existing ASPs, comparing a social-motivational development physical activity program to a health curriculum active control. A total of 10 ASPs in Year 1 and 2 were randomly sampled with stratification criteria that ensured the ASP sampling pool represented underserved communities (enrollment includes at least 50% of youth from low-income households, defined by free/reduced lunch status, and of minority status). From the resultant stratified sampling pool, 5 sites were randomly assigned to the experimental condition to offer adolescents the PA social-motivational climate intervention curriculum. Given the purpose of the study, only data from the intervention condition were analyzed and reported in which the social-motivational climate was implemented as part of the intervention. A repeated measures design was used for the current study to test whether the intervention has the potential to cause change. The Connect through PLAY intervention was administered three days a week for 16 weeks. The baseline data were collected prior to the intervention, the midpoint data were collected at the middle of the intervention (8-week), and the endpoint data were collected following the completion of the intervention. The data collection protocols and data protection procedures, along with parent consent forms and student assent forms, were approved by the University (masked) Internal Review Board prior to data collection.

Given the particularly heightened need and orientation toward social connection, social feedback, and social self-preservation that is characteristic of early adolescence, the intervention was novel in that it focused on facilitating key social mechanisms to support youth self-determined motivation including: (a) building friendships (supporting PA competence, autonomy, and relatedness through encouraging close, mutual relationships with others); (b) promoting group belonging (supporting feelings of being liked and accepted by peers and of being a valued member/contributor to the program, through the establishment of positive peer PA norms, inclusiveness, and tangible PA social support); and (c) fostering staff-youth connections (feeling connected to, supported, and encouraged by staff; see Authors Masked).

## Measures

### Physical activity

Adolescents’ daily MVPA was measured using the Actigraph GT3X (ActiGraph LLC). The participants were assigned to wear the accelerometer on their non-dominate wrist for 7 consecutive days for three times (baseline, midpoint, and endpoint) and to remove the device only for water-based activities (e.g., swimming, bathing). The data files were downloaded using ActiLife Software with 10s sampling interval (Version 6.13.3; ActiGraph LLC). Accelerometer data were considered valid only if over 480 min (8h) of recorded data per day for at least 3 days were present [39]. Then R software was used to read and summarize the acceleration data using the GGIR package with the Euclidean Norm Minus One metric and no imputation [40]. Thresholds developed by Hildebrand et al. [41] were used, with minutes of moderate PA was defined as an average acceleration between 201 and 707 mg and vigorous PA as an average acceleration > 707 mg.

### Intrinsic motivation for physical activity

Adolescents’ intrinsic motivation at baseline and endpoint was assessed by a subscale from the Intrinsic Motivation Inventory (IMI) at the baseline and endpoint [42]. Previous findings have indicated adequate predictive validity and adequate concurrent validity of the IMI Scale with similar demographic samples [43, 44] and has been widely used across work, educational, health, recreational and competitive sport settings [45–47]. The interest/enjoyment subscale consisted of 9 items and a 6-point scale was used to score the participants’ intrinsic motivation level for each item: 1 = very false; 2 = mostly false; 3 = somewhat true; 4 = mostly true; 5 = very true. An example item is “When I am active, I enjoy it.” The scale focused on assessing adolescents’ intrinsic interest and enjoyment. Based on the current sample, the internal consistency reliability coefficients ranged from .93 to .94.

### Autonomous extrinsic motivation

Adolescents’ autonomous extrinsic motivation was assessed using a subscale from the Treatment Self-Regulation Questionnaire [48] at the baseline and endpoint. The autonomous extrinsic motivation subscale consisted of 6 items was used to score the participants’ autonomous extrinsic motivation level; a 5-point Liker scale was used for each item: 1 = strongly disagree, 2 = disagree; 3 = sometimes; 4 = agree; 5 = strongly agree. An example item is “Because I feel that I want to take responsibility for my own health.” Previous findings have indicated adequate predictive validity and adequate concurrent validity of the subscale of Treatment Self-Regulation Questionnaire with similar demographic samples [49, 50]. Based on the current sample, the internal consistency reliability coefficient ranged from .85 to .90.

## Statistical analysis

First, the regression-residual adjustment method [51] was used to calculate self-determination motivations change scores. The calculation is based on regressing all participants’ pre-motivation scores on the post-motivation scores. Each participant’s residual motivation change scores were calculated by subtracting the predicted post-motivation scores from the actual post-motivation scores [51]. A descriptive analysis (means and SDs) was conducted to examine data distributions. Regression analyses were conducted to determine the impact of baseline self-determination motivations, gender, and race on baseline daily MVPA. Then, the intraclass correlation coefficient (ICC) was computed to determine if the equal variance assumption of the motivational variables was satisfied between schools. Lastly, because adolescent daily MVPA across the three measurement points was nested in individual participants, a two-level hierarchical linear model (HLM) was built and tested to answer the three research questions. HLM describes the growth curve of each individual and pieces these curves together to estimate an overall daily MVPA change trajectory [52]. The quadratic model was used for the level 1 model because the plot graph, depicting the relationship between time and MVPA, was not a linear/straight line [52].

The ICC results for changes in intrinsic motivation was .08, and for autonomous extrinsic motivation changes it was .09. These results indicated that between-school variability accounted for a maximum of 9% of the variance in adolescents’ scores for the motivation variables. Based on the criteria set by Murray [53], an ICC < .10 suggests that including individual scores in the level-2 model is recommended. Given the ICCs were less than .10 and that the purpose of this study was to examine the individual’s daily MVPA changing trajectory during a 16-week intervention, it was determined appropriate to use the individual score in the level-2 model. The HLM models have substantial application for the longitudinal data where repeated measures varying across time are nested within individual data [54, 55]. The purpose of the first research question was to examine the average changing trajectory of adolescents’ daily MVPA across the 16-week intervention and the influences of gender, and race on the changing trajectories. Model A (no variable was included in the Level-2 model) and model B (gender and race were included in the level-2 model), as shown below, were constructed and tested to answer the first research question.

Model A:

Level-1 model:

$${\mathrm{MVPA}}_{\mathrm{ti}}= {\uppi }_{0\mathrm{i}}+ {\uppi }_{1\mathrm{i}}* ({\mathrm{TIME}}_{\mathrm{ti}}) + {\mathrm{e}}_{\mathrm{ti}}$$

Level-2 model:

$${\uppi }_{0\mathrm{i}}= {\upbeta }_{00}+ {\Upsilon }_{0\mathrm{i}}$$

$${\uppi }_{1\mathrm{i}} = {\upbeta }_{10} +{\Upsilon }_{1\mathrm{i}}$$

Model B:

Level 1 model:

$${\mathrm{MVPA}}_{\mathrm{ti}} = {\uppi }_{0\mathrm{i}} + {\uppi }_{1\mathrm{i}} * ({\mathrm{Time}}_{\mathrm{ti}}) + {\mathrm{e}}_{\mathrm{ti}}$$

Level 2 model:

$${\uppi }_{0\mathrm{i}} = {\upbeta }_{00} + {\upbeta }_{01} * (\mathrm{Gender}) + {\upbeta }_{02} * (\mathrm{Race}) + {\Upsilon }_{0\mathrm{i}}$$

$${\uppi }_{1\mathrm{i}} = {\upbeta }_{10} + {\upbeta }_{11} * (\mathrm{Gender}) + {\upbeta }_{12} * (\mathrm{Race}) +{\Upsilon }_{1\mathrm{i}}$$

The purpose of the second research question was to examine the influences of intrinsic interest change, and autonomous extrinsic motivation change on the changing MVPA trajectories. Thus, the intrinsic interest change, and autonomous extrinsic motivation change were included in the level-2 model. Model C, as shown below, was constructed and tested to answer the second research question.

Model C:

Level 1 model:

Level 1 model: $${\mathrm{MVPA}}_{\mathrm{ti}} = {\uppi }_{0\mathrm{i}} + {\uppi }_{1\mathrm{i}} * ({\mathrm{Time}}_{\mathrm{ti}}) + {\mathrm{e}}_{\mathrm{ti}}$$  

Level 2 model:

$${\uppi }_{0\mathrm{i}} = {\upbeta }_{00} + {\upbeta }_{01}* (\mathrm{intrinsic\,interest\,change}) + {\upbeta }_{02} * (\mathrm{autonomous\,extrinsic\,motivation\,change}) + {\Upsilon }_{0\mathrm{i}}$$

$${\uppi }_{0\mathrm{i}} = {\upbeta }_{00} + {\upbeta }_{11} * (\mathrm{intrinsic\,interest\,change}) + {\upbeta }_{12} * (\mathrm{autonomous\,extrinsic\,motivation\,change}) + {\Upsilon }_{1\mathrm{i}}$$

In Model A, B, and C, the time variable was centered on the first measurement of adolescent daily MVPA. In other words, the first time point was coded as 0. In Model A, B, and C, π_0i_ represents adolescents’ daily MVPA levels at the beginning of the intervention, while π_1i_ represents the acceleration of the changing rate of adolescents’ daily MVPA over 16 weeks [52]. The HLM analyses were conducted in the HLM software (Version 7.0, Skokie, IL).

## Results

The descriptive results for daily MVPA, as shown in Table 1, suggest that on average adolescents’ daily MVPA tended to increase over the 16-week intervention. The descriptive results for self-determination motivations, as shown in Table 2, indicate that adolescents’ intrinsic motivation increased over the 16-week intervention. Chi-Squared tests showed that there were no significant differences for intrinsic motivation [*χ*^*2*^ (12, 193) = 15.47, *p* = .22; *χ*^*2*^ (71, 193) = 78.39, *p* = .26] and autonomous extrinsic motivation [*χ*^*2*^ (19, 193) = 14.28, *p* = .77; *χ*^*2*^ (88, 193) = 93.99, *p* = .31] between boys and girls at baseline and in the residual change scores, respectively.

**Table 1:** Descriptive statistics for daily MVPA

	Total		Male		Female	
Variables	Mean/SD	Skew/Kurt	Mean/SD	Skew/Kurt	Mean/SD	Skew/Kurt
Baseline MVPA (min)	43.21/27.79	.75/1.172	47.57/29.12	1.17/2.24	36.43/35.92	.98/1.14
Midpoint MVPA (min)	53.73/25.36	.66/1.08	61.80/23.32	.33/.66	46.80/23.33	.42/-.25
Endpoint MVPA (min)	54.41/26.70	.14/-.64	64.01/26.14	-.06/-.33	46.38/24.56	.31/-.57

*Abbreviations*: *MVPA* moderate-to-vigorous physical activity, *SD* Standard Deviation

**Table 2:** Descriptive statistics for self-determination motivation variables

	Intrinsic motivation	Autonomous extrinsic motivation
	Mean/SD	Mean/SD
Baseline	3.88/.71	4.36/.97
Endpoint	3.92/.77	4.17/1.06

*Abbreviation*: *SD* Standard Deviation

The regression analyses results showed that the overall regression model was significant, *F* (6,112) = 2.309, *p* =.039, *R*^*2*^=.12. Gender (*β* = .57, *t* = 3.85, *p* < .001) predicted adolescent’s daily MVPA, while race, baseline intrinsic interest, and baseline autonomous extrinsic motivation were not significant predictors (*β* = -3.36, *t* = -1.77, *p* = .08; *β* = 8.74, *t* = 1.94, *p* = .055; *β* = -3.24, *t* = -1.13, *p* = .26). These findings indicate that boys had higher daily MVPA than girls at baseline.

## HLM results

Tables 3 and 4 show the results of the HLM analysis of Model A, B and C. The fixed part of the intercept describes the overall mean scores of daily MVPA at baseline, while the random part describes the heterogeneity of scores around the intercept mean. Specifically, Model A results indicated that the average daily MVPA at baseline was 43.21 minutes, but there was heterogeneity around the mean. On average, youth daily MVPA increased 6.36 minutes in each 8-week period (coefficient = 6.36, *p* < .01). The random part of the quadratic term (π_1_) was significant (*SD* = 5.38, *p* = .008) which indicated different students demonstrated different daily MVPA changing trajectories over the 16-week intervention. Adolescents who had higher daily MVPA at baseline tended to have a slower (*π*_*1*_ = -.256) changing trajectory than adolescents who had lower daily MVPA at baseline. The results of Model B indicated that gender (coefficient = -4.06; *p* = .067) and race (coefficient = .21, *p* = .79) were not significant predictors of daily MVPA changes. The HLM analysis of Model C identified intrinsic motivation change (coefficient = 2.57; *p* = .035), but not autonomous extrinsic motivation change, as a significant level-2 predictor of daily MVPA changes.

**Table 3:** Estimation of fixed effects of model A, B and C

Fixed effect	Coefficient	*p* value
Estimation of Fixed Effects of Model A		
For the intercept1, π_0_		
Intercept 2, β_00_	43.21	< .001
For time slope, π_1_		
Intercept 2, β_10_	6.36	< .001
Estimation of Fixed Effects of Model B		
For the intercept1, π_0_		
Intercept 2, β_00_	170.53	< .001
Gender, β_01_	-12.79	.002
Race, β_02_	-1.29	.322
For time slope, π_1_		
Intercept 2, β_10_	-5.42	.659
Gender, β_11_	-4.06	.067
Race, β_12_	.21	.79
Estimation of Fixed Effects of Model C		
For the intercept1, π_0_		
Intercept 2, β_00_	48.31	< .001
Intrinsic interest, β_01_	7.71	.002
Autonomous extrinsic motivation, β_02_	-1.48	.545
For time slope, π_1_		
Intercept 2, β_10_	2.91	.002
Intrinsic interest, β_11_	2.57	.035
Autonomous extrinsic motivation, β_12_	.86	.473

**Table 4:** Estimation of random effects of model A, B and C

Random effect	SD	Variance	*p* value
Estimation of Random Effects of Model A			
Intercept1, ϒ_0_	23.48	551.30	< .001
Time slope, ϒ_1_	5.38	28.93	.008
Level 1, *e*	13.64	185.97	
Estimation of Random Effects of Model B			
Intercept1, ϒ_0_	19.15	366.67	< .001
Time slope, ϒ_1_	7.47	55.73	< .001
Level 1, *e*	11.00	121.04	
Estimation of Random Effects of Model C			
Intercept1, ϒ_0_	21.93	480.90	<.001
Time slope, ϒ_1_	7.23	52.34	< .001
Level 1, *e*	11.01	121.15	

## Discussion

This study set out to examine changes in the MVPA of at-risk adolescents participating in a 16-week PA social-motivational climate-based intervention within ASPs, with specific focus on whether changes in self-determination motivations leads to increases in daily PA engagement. Therefore, the following research questions were addressed: (a) to what extent does adolescents’ daily MVPA change from baseline to endpoint during a 16-week SDT-based intervention within ASPs, and does this change vary by gender and race? And (b) to what extent does change of adolescent intrinsic and autonomous extrinsic motivations across the 16-week intervention influence the trajectory of their daily MVPA over the 16-week period. There were three key findings from this study. First, adolescents’ daily MVPA was found, on average, to increase by 12.72 minutes from baseline to endpoint. Second, despite clear gender and race differences in youth MVPA at baseline, gender and race were not significant predictors of adolescents’ daily MVPA change trajectories, suggesting that across gender and race, youth had a similar rate of change in MVPA across the 16-week intervention. Third, change in intrinsic motivation, but not autonomous extrinsic motivation, was a significant predictor of adolescents’ daily MVPA changes.

Although there are significant benefits of MVPA to the health and well-being of youth [2] and ASPs are an ideal setting to support youth recommended daily PA accrual, several ASPs intervention studies that have aimed to increase adolescent MVPA through improving the motivational climate have reported a lack of adequate daily MVPA and challenges with maintaining children’s attendance over time [56, 57]. In a previous empirical study, grounded in SDT, the Active Play Trial emphasized the necessity of retaining the self-chosen and child-managed character of play, as well as supporting children’s need for competence, relatedness, and autonomy [56]. The Active Play Trial showed no significant intervention effects were observed for children’s MVPA in ASPs compared with control programs [56]. The Active by Choice Trial integrated principles from SDT that aimed to test the efficacy of a motivational plus behavioral skills intervention on increasing MVPA in underserved adolescents [57]. The findings showed a nearly 5-minute increase in daily MVPA at midpoint, but these results were not sustained at 2-weeks post-intervention. In our current study, adolescents, on average, were accumulating 43.21 minutes per day at baseline and close to the recommended 60 min of daily MVPA (54.41 min) at endpoint. The results showed not only that adolescents’ daily MVPA increased but also that this increase was sustained over the 16-week intervention period. The findings provide preliminary but significant evidence suggesting a benefit of integrating SDT-based approaches (autonomy and relatedness support) in under-resourced ASPs. This result aligns with Cox’s et al. [58] notion that facilitating a motivational climate that fosters self-determined motivations can result in sustained improvements throughout the intervention in youth daily MVPA.

Although baseline regression analyses and HLM Model C clearly showed that youth baseline intrinsic and autonomous extrinsic motivations did not significantly predict baseline daily MVPA, the change in intrinsic motivation was observed as a significant positive predictor while the change in autonomous extrinsic motivation was not a significant predictor. The results may shed new light on our understanding of the role of intrinsic and autonomous extrinsic motivations change. The observed increase in intrinsic motivation indicates that facilitating a socially-based SDT PA-climate may improve and maintain adolescents’ intrinsic motivation to participate into MVPA over time. The results support the notion that intrinsic motivation supports the most desirable and long-lasting levels of engagement [21, 44, 59]. The observed slight decrease in autonomous extrinsic motivation is worth future examination and may be due to several reasons. One primary possibility is that decreases in autonomous extrinsic motivation reflect youth moving increasingly towards intrinsic motivation throughout the intervention. Compared with identified motivation and integrated motivation, intrinsic motivation involves the highest degree of autonomous behaviors and fosters engagement in an activity for the inherent feeling of enjoyment, a personal sense of accomplishment, and/or the experience of learning new things [21]. The current intervention program has a special focus on increasing adolescents’ intrinsic motivation by both establishing an inclusive and engaging climate that supports guided autonomy (e.g., incorporates youth choice and ownership/empowerment over activities offered) and offering collaborative and cooperative PA activities that emphasize teamwork, mastery, and playful enjoyment (See Authors Masked). The findings further suggest that nurturing intrinsic motivation can be a highly effective approach to supporting youth MVPA.

Furthermore, ASPs should tailor their programming in ways that provide equal access, and a wide range of PA opportunities that appeal to and support all adolescents across age, gender, race/ethnicity, and family socioeconomic status [29]. In particular, girls worldwide consistently report significantly lower levels of PA than do boys and show steeper declines in PA from childhood to adolescence [36]. The prevalence rates for inactivity are particularly high for low-income girls of color who typically have the least PA opportunities [60]. Interventions and other PA initiatives that have shown any kind of success at improving adolescents’ PA, have reported improvements predominately in boys PA, with markedly less improvement in girls PA [36]. With this understanding, ASP designers and educators should provide active choices that are based on the needs and interests of girls and minority youth in order to reduce the persistent disparities observed in youth PA [36]. In the current study, gender differences in MVPA, where boys accrued significantly more daily MVPA than girls, were observed at baseline reflecting the persistent historical gender disparities found in PA. However, the results of HLM model B indicated that gender and race were not significant predictors of daily MVPA changes in the 16-week intervention. Contrary to challenges of previous PA interventions, the current study indicates that the Connect intervention may be equally supportive of improving both boys and girls daily MVPA. The findings may be explained by the power of the Connect intervention curriculum that follows pedagogical implications of SDT. Along with the need for competence and autonomy, SDT highlights the need for positive social interactions for improving long-term lifestyle changes. According to SDT, the basic human need for social connection and meaningful interpersonal relationships should stimulate goal-directed behaviors to satisfy it [20]. The current intervention, which places an emphasis on supporting youth relatedness needs through fostering youth friendships, positive adult-peer relations, and a strong sense of belongingness, along with nurturing competence and autonomy, suggesting that ASPs can effectively increase girls’ MVPA and may be a critical context for reducing persistent gender disparities in PA when they provide high quality programming. High quality ASPs that provide intentional SDT-based approaches for fostering a positive PA social-motivational climate are needed to improve the health outcomes of underserved youth and thus minimize these inequities [32].

This study has three limitations. First, staff variables (e.g., staff interaction with adolescents, staff motivation, and staff attitude) and social development variables (e.g., social affiliation, social support) are not integrated into this study. Future studies are needed to examine the impact of staff’s support of adolescents’ basic psychological needs for autonomy, competence, and relatedness and social development variables on adolescents’ MVPA changing trajectory. Secondly, given this study did not include the active comparison group, future research with the full randomized controlled trial sample upon the completion of the 5-year intervention should ultimately be used to determine the effects of social-motivational climate on youth MVPA change. Thirdly, although controlled motivation was not measured as part of the study, future research should examine youth change in motivation as a continuum across regulatory orientations throughout an SDT-based intervention, with the goal of youth movement towards more autonomous, and ultimately, intrinsic motivation.

## Conclusion

The current study presents a picture of how an SDT-related motivational climate in ASPs impact adolescents’ MVPA changes over a 16-week intervention. Current findings are believed to be one of the first to identify a positive MVPA changing trajectory among underserved adolescents during a 16-week ASP intervention regardless of their gender and race. The findings suggest that changes in youth intrinsic motivation serves as a critical mechanism by which supportive PA climates promote adolescents’ daily MVPA. Future studies are needed to examine intrinsic and autonomous extrinsic motivations as mediators to better understand the impact of SDT-related motivational mechanism on adolescents’ MVPA.

## Data Availability

A copy of survey instrument questions will be provided upon request to the corresponding author. Participant data are not publicly available due to human subject research protections but qualified academic researchers may send data requests to the corresponding author for review. All use of data would be subject to confidentiality and data-use agreements.

## Abbreviations

SDT: Self-Determination Theory
MVPA: Moderate-to-vigorous physical activity
PLAY: Positive Leisure Activities for Youth
PA: Physical activity
ASPs: Afterschool programs
PE: Physical education
SD: Standard Deviation
ICC: Intraclass correlation coefficient
HLM: Hierarchical linear model
